# Canonical correlation analysis of depression and anxiety symptoms among college students and their relationship with physical activity

**DOI:** 10.1038/s41598-023-38682-w

**Published:** 2023-07-17

**Authors:** Lili Li, Peng Wang, Shufan Li, Qing Liu, Fen Yu, Zhaohui Guo, Shuqi Jia, Xing Wang

**Affiliations:** 1 Shanghai University of Engineering Science, Shanghai, 201620 China; 2 Shanghai University of Sport, Shanghai, 200438 China

**Keywords:** Psychology, Health care

## Abstract

To explore the association between depression and anxiety symptoms among college students and the relationship between the two and physical activity. A cross-sectional study design was used to survey 1790 enrolled university students using the Depression Self-Rating Scale, Anxiety Self-Rating Scale and Physical Activity Rating Scale. 37.75% of male students and 39.73% of female students detected depressive symptoms, 17.65% of male students and 17.86% of female students detected anxiety symptoms, 11.89% of male students and 11.75% of female students detected both depressive and anxiety symptoms. Canonical correlation between depression and anxiety symptoms of college students were significant. The depression and anxiety score of college students in the high level group was significantly lower than that in the low and medium level groups, and no significant difference was found between the low and medium level groups. Affective disorder and anxious mood of male students correlated most closely with intensity, while somatic disorder, psychomotor disorder and depressive psychological disorder correlated most closely with duration. Affective disorder of female students correlated most closely with frequency, depressive psychological disorder and anxious mood correlated most closely with intensity, while premonition of misfortune and frequent urination correlated most closely with duration. Depression and anxiety symptoms of college students were closely related and co-occurrence was common. Students with high level of physical activity had milder symptoms. Different exercise interventions are recommended for different symptoms.

## Introduction

Depression and anxiety are the most common psychological problems in Chinese college students, with the detection rates of 34% and 31%^1^. Depressive symptoms are mainly on depressed mood, accompanied by slow thinking, pessimistic attitude, lack of energy and the reduction of the will activity^2^, while anxiety symptoms were often manifested as irritability, physical tension and sleep disorders^3^, both of which could cause low learning efficiency of students and difficulties in interpersonal communication, and were highly correlated with autotomy, which was the high risk factor for suicide^4,5^. According to the Chinese survey, 69% of depressed patients also suffered from anxiety disorder^6^. Compared with the single mental disorder, depressive and anxiety comorbidities had more severe symptoms, higher suicide rate and worse prognosis^7^. Therefore, it was urgent to further the research on depression and anxiety in colleges and universities, and it was necessary to identify and intervene both depression and anxiety in colleges and universities. Canonical Correlation Analysis (CCA) can examine the overall linear correlation between the two sets of variables^8^, providing a new way of thinking about the simultaneous study of depression and anxiety symptoms.

Evidence showed that physical exercise was an effective means to promote mental health, with some advantages such as stable effects, high compliance and few side effects^9^. Cross-sectional studies showed that high physical activity level was a protective factor for depression and anxiety symptoms of college students^10^. Longitudinal study confirmed that after 6-week regular exercise, self-rating scale scores of depression and anxiety of college students decreased significantly^11^. Neurobiological studies have found that exercise can increase cerebral blood flow perfusion^12^, up-regulate the level of neurotrophic factors^13^, improve the function of hypothalamic–pituitary–adrenal axis^14^, inhibit the secretion of proinflammatory cytokines^15^, effectively stimulate the central nervous system, and then relieve depression, anxiety and other negative emotions^16^.

Reviewing the previous literature, we knew that physical exercise could effectively improve the symptoms of depression and anxiety in college students, and only by identifying the relationship between symptoms can we ensure the "right medicine" and the accuracy of the exercise program (exercise intensity, duration, frequency, etc.). Therefore, this study intends to probe the following questions: what is the relationship between depression and anxiety among college students? Which symptoms will appear simultaneously? What is the correlation between the elements of physical exercise and depression and anxiety? What kind of physical exercise habits do students with the mildest symptoms have? Based on the above problems and previous studies, this study aims at exploring the correlation between depression and anxiety symptoms among college students and analyzing the relationship between the main symptoms and physical exercise, in order to provide directions and ideas for clinical identification and targeted intervention of depression and anxiety symptoms among college students, and to provide references for scientific research workers and university managers.

## Research object and research method

### Subject recruitment

The study was conducted from September to December 2022, in the midst of the COVID-19 pandemic, when infected students needed to be isolated. All participants were enrolled in university, aged from 18 to 24 years, without psychiatric disorders, without exercise contraindications such as respiratory tract, cardiovascular and cerebrovascular diseases, and without taking psychotropic drugs such as barbiturates, benzodiazepines and chloral hydrate before. All participants participated in the test voluntarily and all of them signed informed consent. This research was approved by the University Ethics Committee (102772021RT004) and all methods were performed in accordance with the relevant guidelines and regulations.

A total of 1823 university students were recruited to volunteer for this study in seven universities in Songjiang University City, Shanghai, to whom questionnaires were distributed. Before the participants filled in, the investigators read out instructions and explanatory entries, clarifying that the data obtained were only used for scientific research, emphasizing true, independent and voluntary responses, and informing participants of their right to quit midway. During the filling process, the participants were prompted to answer carefully according to the requirements; After the participants filled in, the investigators would check the missing items and the filling contents contrary to common sense, and ensure the completeness, accuracy and authenticity of the information by means of filling and refilling. What’s more, the investigators excluded questionnaires that took less than 3 min to complete and questionnaires that were answered regularly.

### Tools for test

#### Basic information table

The subjects were asked about their gender, age, height, weight and other basic information, as well as whether they had mental illness and exercise contraindications.

#### Self-rating depression scale (SDS)

This questionnaire was compiled by Professor Zung of Duke University. At present, it has been widely used in the screening of depression tendency of college students, reflecting the depressive mood of the subjects in the past week^17^, with a total of 20 items divided into four dimensions: Psychomotor disorders (item 1, 3), somatic disorders (item 2, 4, 5, 6, 7, 8, 9, 10), psychomotor disorders (item 12, 13), and depressive psychological disorders (item 11, 14, 15, 16, 17, 18, 19, 20)^18^. The options for each item were "no or little time", "little time", "considerable time" and "most time", which were calculated from 1 to 4 points respectively. The forward score and the reverse score were 10 questions respectively. The SDS score was the sum of the scores of each question, multiplied by 1.25 and rounded, and the score ranged from 25 to 100 points. According to the results of Chinese norm, ≥ 53 points indicated depressive tendency. In this study, the Cronbach's α coefficient of this scale was 0.87.

#### Self-rating anxiety scale (SAS)

This questionnaire was compiled by Professor Zung of Duke University. It reflected the anxiety of the subjects in the past week^19^, with a total of 20 items divided into four dimensions: Anxious mood (items 1, 2, 3, 4), vegetative nervous disorder (items 7, 8, 10, 11, 12, 14, 15, 18), motor tension (items 6, 9, 13, 17, 19, 20), premonition of misfortune and frequent urination (items 5, 16)^20^. The options for each item were "no or little time", "little time", "considerable time" and "most time", which were calculated from 1 to 4 points respectively. The forward score and the reverse score were 10 questions respectively. The SDS score was the sum of the scores of each question, multiplied by 1.25 and rounded, and the score ranged from 25 to 100 points. According to the results of Chinese norm, ≥ 50 indicated anxiety tendency. In this study, the Cronbach's α coefficient of this scale was 0.93.

#### The physical activity scale-3 (PARS-3)

This questionnaire was revised into Chinese by Liang Deqing et al., Wuhan Institute of Physical Education, and was currently recognized as one of the effective questionnaires for measuring adult physical activity level^21^, with a total of 4 items. Participants were asked about the intensity, duration, frequency and type of their regular exercise. This scale defined the amount of exercise = intensity × time × frequency, in which intensity and frequency were divided into 5 grades, 1 ~ 5 points respectively, and the time were divided into 5 grades, 0 ~ 4 points respectively, and the score ranged from 0 to 100 points. Evaluation criteria: ≤ 19 was classified as small amount of exercise, 20 ~ 42 was classified as moderate amount of exercise, ≥ 43 was classified as large amount of exercise. In this study, the Cronbach's α coefficient of this scale was 0.74.

### Data analysis

All statistical analyses were performed based on SPSS 26.0. All parameters were statistically inferred, using a two-tailed test with the test level α set at 0.05. The independent sample t test was used to compare the symptoms of depression, anxiety and physical exercise of male and female college students. Pearson correlation coefficient analysis was used to explore the relationship between depression and anxiety symptoms among university students. Canonical correlation analysis was used to extract statistically significant pairs of variables, use their correlation coefficients to measure the degree of relationship between typical variables, and then examine the degree of contribution of the variables on their ipsilateral typical variables as measured by their loadings. One-way ANOVA and LSD post-hoc multiple tests were used to compare depression and anxiety symptoms among university students with different levels of physical activity. Logistic regression analysis was used to explore the relationship between physical activity (exercise intensity, exercise frequency, exercise duration) and depression and anxiety symptoms.

## Results

### Basic characteristics of subjects included

A total of 1,811 students filled in the questionnaire, and 1790 of them were included in the data analysis, excluding 13 students who dropped out, 5 students who took less than 3 min to complete the questionnaire, and 3 students who responded regularly (consistent responses throughout). The gender ratio was basically balanced, including 816 male students (45.59%) and 974 female students (54.41%). The overall age was 19.83 ± 1.69 years old, and the BMI was 21.20 ± 3.63 kg/m^2^. What is more, 58.67% were only children and 66.41% were urban residents.

**Figure 1:**
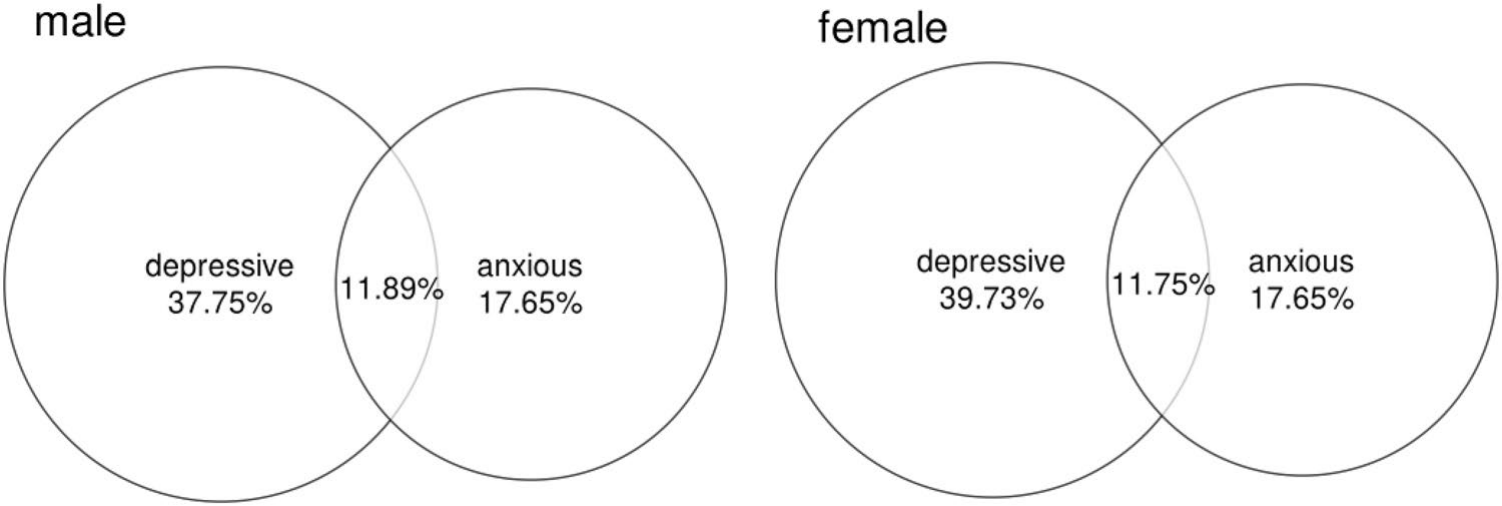
The comorbidity relationship between depression and anxiety symptoms among college students (N = 1790).

As shown in Fig. 1, 37.75% of male students and 39.73% of female students were found to have depressive symptoms, the scores of all dimensions of the self-rating scale for male students were significantly lower than those of female students (*P* < 0.05). 17.65% of male students and 17.86% of female students were found to have anxiety symptoms, and the score of the exercise tension (A3) of the self-rating scale for male students was significantly lower than that of female students (*P* < 0.05). What’s more, 11.89% of male students and 11.75% of female students were found to have depression and anxiety symptoms at the same time. As shown in Table 1, the physical exercise level of male students was significantly higher than that of female students in terms of intensity, duration and frequency (*P* < 0.001). In this study, college students of different genders were analyzed separately to reduce results bias.

**Table 1:** Basic characteristics of included subjects (N = 1790).

Variables	Male (n = 816)	Female (n = 974)	Comparison among groups	
			t value	*P* value
SDS	46.66 ± 10.99	48.23 ± 9.62	− 3.185	0.001
Affective disorder	2.90 ± 1.12	3.01 ± 1.05	− 2.170	0.030
Somatic disorder	13.98 ± 3.42	14.30 ± 3.02	− 2.088	0.037
Psychomotor disorder	4.06 ± 1.30	4.20 ± 1.18	− 2.420	0.016
Depressive psychological disorder	16.40 ± 5.17	17.08 ± 4.76	− 2.874	0.004
SAS	41.29 ± 9.36	41.42 ± 7.88	− 0.303	0.762
Anxious mood	5.57 ± 2.11	5.45 ± 1.98	1.256	0.209
Vegetative nervous disorder	10.72 ± 3.57	10.56 ± 2.92	1.034	0.301
Motor tension	12.68 ± 3.50	13.00 ± 3.25	− 1.969	0.049
Premonition of misfortune & Frequent urination	4.06 ± 1.31	4.13 ± 1.26	− 1.101	0.271
PARS-3	31.21 ± 24.15	17.74 ± 17.13	13.355	< 0.001
Exercise intensity	2.82 ± 1.25	1.98 ± 1.08	15.091	< 0.001
Exercise duration	2.76 ± 1.29	2.46 ± 1.22	4.994	< 0.001
Exercise frequency	3.37 ± 1.00	3.10 ± 0.94	5.936	< 0.001

### Relationship between depression and anxiety symptoms of college students

Pearson correlation analysis was conducted on depression and anxiety symptoms of college students. The results were shown in Table 2: all symptoms of depression and anxiety of male college students were significantly positively correlated (*P* < 0.01), At the same time, most of the depression and anxiety symptoms of female college students had a significant correlation, which provided the basis for the follow-up typical correlation analysis.

**Table 2:** Correlation coefficient matrix of depression and anxiety symptoms (N = 1790).

Gender	Depression	Anxiety			
		Anxious mood	Vegetative nervous disorder	Motor tension	Premonition of misfortune & Frequent urination
Male (n = 816)	Affective disorder	0.614	0.457	0.108	0.152
	Somatic disorder	0.427	0.383	0.428	0.398
	Psychomotor disorder	0.392	0.311	0.444	0.431
	Depressive psychological disorder	0.168	0.155	0.591	0.532
Female (n = 974)	Affective disorder	0.604	0.485	0.044	0.144
	Somatic disorder	0.319	0.385	0.295	0.327
	Psychomotor disorder	0.311	0.240	0.301	0.343
	Depressive psychological disorder	0.054	0.047	0.518	0.538

Canonical correlation analysis was used to explore the relationship between the two groups of symptom dimensions of depression and anxiety, as shown in Table 3. Four pairs of canonical variables were extracted from each group. Among male college students, the first two pairs of canonical variables showed significant (*P* < 0.001), and the canonical correlation coefficients were 0.733 and 0.574, respectively, which were included in the follow-up analysis. Among female college students, the first three pairs of canonical variables showed significance (*P* < 0.001), and the canonical correlation coefficients were 0.675, 0.603 and 0.195, respectively. In view of the low coefficient of the third pair, only the first two pairs would be analyzed later. For the convenience of analysis, the first pair of typical variables were named D1 and A1, and the second pair were named D2 and A2, in which D was mainly used to extract information of all dimensions of depressive symptoms, and A was mainly used to extract information of all dimensions of anxiety symptoms.

**Table 3:** Typical correlation coefficient and significance test of depression and anxiety symptoms (N = 1790).

Gender	Canonical correlation pair	Canonical correlation coefficient	Test of significance			
			Wilks'	Chi-SQ	F value	*P* value
Male (n = 816)	1	0.733	0.308	2469.12	72.558	< 0.001
	2	0.574	0.665	1969.04	39.887	< 0.001
	3	0.086	0.993	1620.00	1.510	0.197
	4	0.006	1.000	811.00	0.026	0.872
Female (n = 974)	1	0.675	0.333	2951.82	79.817	< 0.001
	2	0.603	0.612	2353.58	58.422	< 0.001
	3	0.195	0.962	1936.00	9.581	< 0.001
	4	0.020	1.000	969.00	0.379	0.538

**Figure 2:**
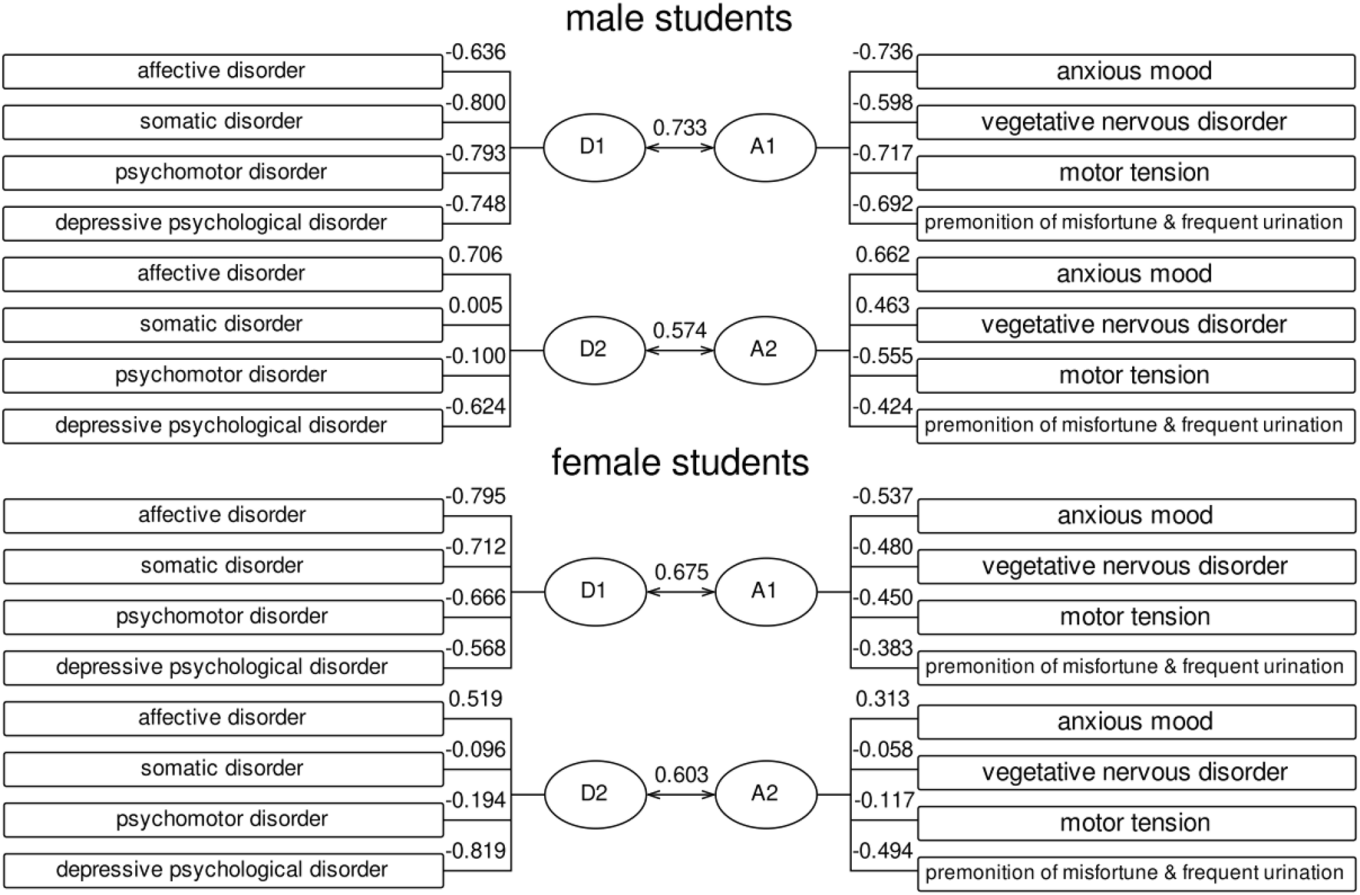
Canonical correlation analysis load coefficient (N = 1790).

As shown in Fig. 2, among male college students, D1 had a higher load coefficient on somatic disorders and psychomotor disorders, D2 had a higher load coefficient on affective disorders, and A1 and A2 both had a higher load coefficient on anxiety, suggesting that male depression symptoms might be synchronized with anxiety. Among female college students, D1 had a higher load coefficient for affective disorders, A1 had a higher load coefficient for anxiety, D2 had a higher load coefficient for depressive psychological disorders, and A2 had a higher load coefficient for premonition of misfortune and frequent urination, suggesting that female students’ affective disorders might be synchronous with anxiety, and depressive psychological disorder coincided with foreboding and frequent urination.

As shown in Table 4, the first typical redundancy (the percentage of the variance of the original variable group explained by the typical variable formed by the original variable group) and the second typical redundancy (the percentage of the variance of the original variable group explained by the typical variable formed by the other party) of male students' depression and anxiety symptoms were respectively 78.29% and 75.87%, 34.82% and 37.39%. The first canonical redundancy and the second canonical redundancy of female students’ depression and anxiety symptoms were 72.30% and 75.69%, respectively, and 31.52% and 30.65%, respectively.

**Table 4:** Explanatory power of typical variables (%) (N = 1790).

Gender	Interpretant	D1	D2	D1 + D2	A1	A2	A1 + A2
Male (n = 816)	SDS	55.87	22.41	78.29	25.42	9.40	34.82
	SAS	30.00	7.39	37.39	47.35	28.52	75.87
Female (n = 974)	SDS	47.63	24.67	72.30	19.87	11.65	31.52
	SAS	21.69	8.96	30.65	43.64	32.05	75.69

These results showed that in college students, regardless of gender, the explanatory power of depression and anxiety symptoms could reach more than 70% when only the first two pairs of typical variables were analyzed. Both of the two pairs of variables could well predict their own typical variable groups, and could predict the corresponding variable groups to a certain extent. Depression and anxiety symptoms were closely related.

### Relationship between depression, anxiety symptoms and physical exercise of college students

CCA showed that the depression and anxiety symptoms of male college students were mainly determined by affective disorder, somatic disorder, psychomotor disorder, depressive psychological disorder and anxious mood, and they were closely related to each other. The symptoms of female college students were mainly determined by affective disorder, anxious mood, depressive psychological disorder, premonition of misfortune and frequent urination, in which the former two were closely related and the latter two were closely related. Therefore, the relationship between these specific symptoms and physical exercise was analyzed.

**Figure 3:**
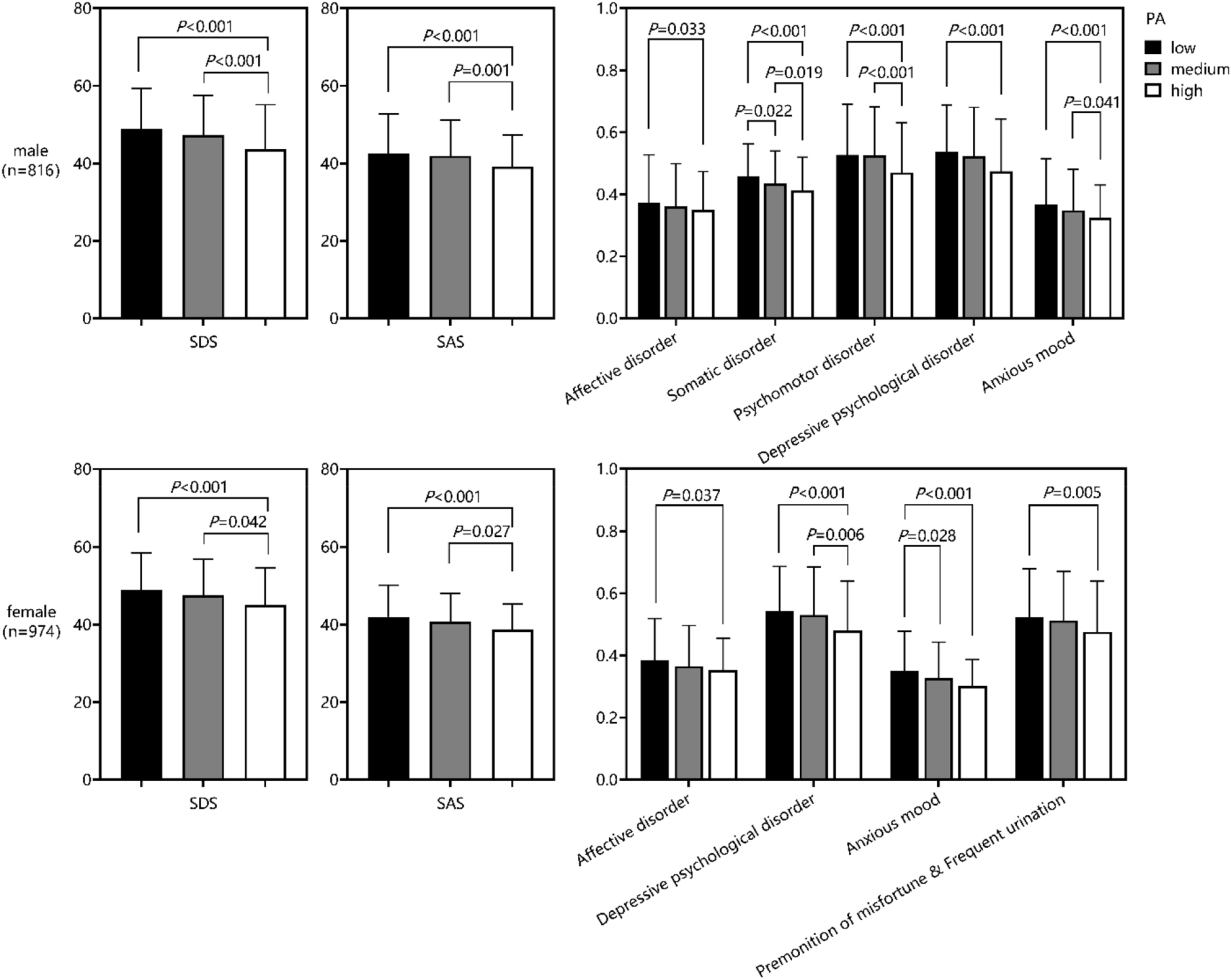
Comparison of symptoms of college students with different levels of physical activity (N = 1790).

As shown in Fig. 3, the depression and anxiety score of college students in the high level group was significantly lower than that in the low and medium level groups, and no significant difference was found between the low and medium level groups. For male students, the scores of all symptoms in the low-level group were significantly higher than those in the high level group (all *P* < 0.05), the scores of psychomotor disorder and anxiety in the low level group were significantly higher than those in the medium level group (all *P* < 0.05) and there were significant differences in somatic disorder among the three groups (all *P* < 0.05). For female students, the scores of all symptoms in the low level group were significantly higher than those in the high level group (all *P* < 0.05), the scores of somatic disorder in the low level group were significantly higher than those in the medium level group (*P* = 0.006) and the scores of anxiety in the medium level group were significantly higher than those in the high level group (*P* = 0.028). See Table 5 for specific scores.

**Table 5:** Comparison of SDS and SAS scores of college students with different levels of physical activity (N = 1790).

Gender	Physical activity	Depression					Anxiety				
		SDS	Affective disorder	Somatic disorder	Psychomotor disorder	Depressive psychological disorder	SAS	Anxious mood	Vegetative nervous disorder	Motor tension	Premonition of misfortune & Frequent urination
Male (n = 816)	Low (n = 331)	48.79 ± 10.47	2.99 ± 1.23	14.62 ± 3.37	4.22 ± 1.30	17.21 ± 4.83	42.61 ± 10.13	5.88 ± 2.35	11.16 ± 3.96	12.85 ± 3.55	4.19 ± 1.32
	Medium (n = 212)	47.20 ± 10.34	2.89 ± 1.10	13.94 ± 3.31	4.20 ± 1.25	16.72 ± 5.06	41.93 ± 9.26	5.58 ± 2.12	10.76 ± 3.49	13.00 ± 3.38	4.21 ± 1.31
	High (n = 273)	43.66 ± 11.47	2.80 ± 0.99	13.22 ± 3.40	3.76 ± 1.28	15.16 ± 5.43	39.20 ± 8.07	5.18 ± 1.71	10.16 ± 3.03	12.23 ± 3.51	3.79 ± 1.25
Female (n = 974)	Low (n = 681)	48.91 ± 9.61	3.07 ± 1.08	14.44 ± 3.10	4.28 ± 1.19	17.35 ± 4.62	42.00 ± 8.15	5.59 ± 2.07	10.77 ± 3.08	13.05 ± 3.22	4.19 ± 1.24
	Medium (n = 194)	47.46 ± 9.31	2.92 ± 1.05	13.96 ± 2.84	4.11 ± 1.10	16.97 ± 4.91	40.78 ± 7.16	5.24 ± 1.84	10.17 ± 2.55	13.11 ± 3.32	4.10 ± 1.26
	High (n = 99)	45.06 ± 9.58	2.83 ± 0.81	13.98 ± 2.77	3.88 ± 1.22	15.36 ± 5.09	38.64 ± 6.66	4.82 ± 1.36	9.88 ± 2.24	12.40 ± 3.27	3.81 ± 1.30

As shown in Table 6, for both male and female students, all symptom scores were negatively correlated with the intensity, duration and frequency of physical exercise, and most of them were significant. Affective disorder and anxious mood of male students correlated most closely with intensity, while somatic disorder, psychomotor disorder and depressive psychological disorder correlated most closely with duration. Affective disorder of female students correlated most closely with frequency, depressive psychological disorder and anxious mood correlated most closely with intensity, premonition of misfortune and frequent urination correlated most closely with duration.

**Table 6:** Correlation between physical activity and symptoms of depression and anxiety in college students (N = 1790).

Gender	Symptoms	Physical activity		
		Intensity	Duration	Frequency
Male (n = 816)	Affective disorder	r = − 0.078 *P* = 0.026	r = − 0.076 *P* = 0.029	r = − 0.046 *P* = 0.187
	Somatic disorder	r = − 0.143 *P* < 0.001	r = − 0.153 *P* < 0.001	r = − 0.150 *P* < 0.001
	Psychomotor disorder	r = − 0.115 *P* = 0.001	r = − 0.129 P < 0.001	r = − 0.128 *P* < 0.001
	Depressive psychological disorder	r = − 0.142 *P* < 0.001	r = − 0.149 *P* < 0.001	r = − 0.148 *P* < 0.001
	Anxious mood	r = − 0.148 *P* < 0.001	r = − 0.145 *P* < 0.001	r = − 0.071 *P* < 0.001
Female (n = 974)	Affective disorder	r = − 0.064 *P* = 0.047	r = − 0.079 *P* = 0.013	r = − 0.086 *P* = 0.007
	Depressive psychological disorder	r = − 0.118 *P* < 0.001	r = − 0.094 *P* = 0.003	r = − 0.101 *P* = 0.002
	Anxious mood	r = − 0.093 *P* = 0.004	r = − 0.083 *P* = 0.009	r = − 0.084 *P* = 0.009
	Premonition of misfortune & Frequent urination	r = − 0.086 *P* = 0.007	r = − 0.087 *P* = 0.006	r = − 0.046 *P* = 0.153

**Figure 4:**
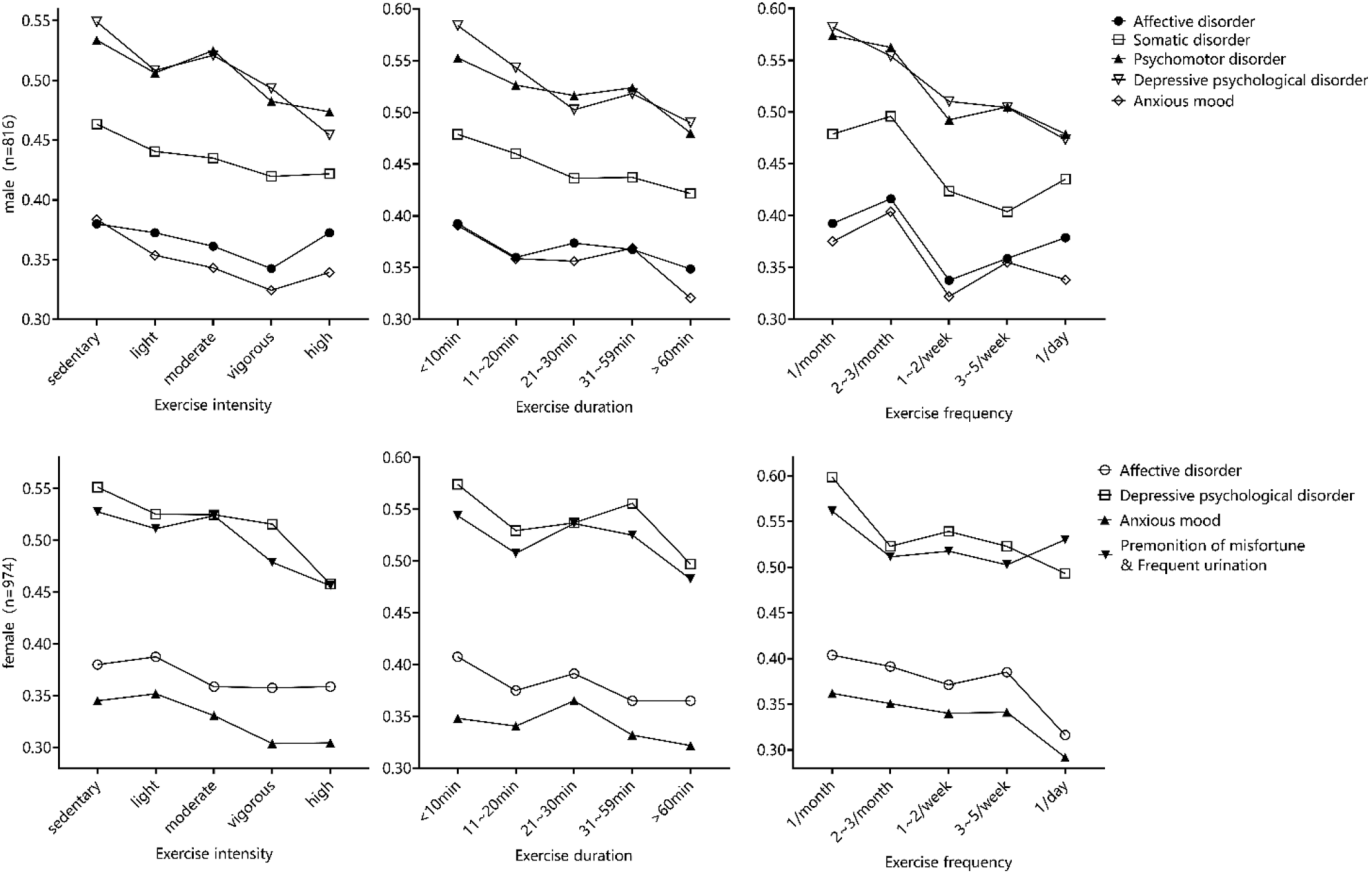
Relationship between physical activity and symptoms of depression and anxiety among college students (N = 1790).

As shown in Fig. 4, male students who exercised at high intensity for 1 h or more per session, once per day had the lowest level of motor tension, premonition of misfortune and frequent urination, who exercised at high intensity for 1 h or more per session, 3–5 times per week had the lowest level of somatic disorder, who exercised at high intensity for more than 1 h per session, 1–2 times per week had the lowest levels of affective disorder and anxious mood. Female students who exercised at high intensity for 31–59 min per session, once per day had the lowest level of affective disorder, who exercised at high intensity for more than one hour once a day had the lowest level of depressive psychological disorder, who exercised at moderate intensity for 31–59 min once a day had the lowest level of anxious mood, while who exercised at high intensity for more than one hour 3–5 times per week had the lowest premonition of misfortune and frequent urination.

## Discussion

This study found that the co-occurrence of depression and anxiety symptoms was common among college students, and the detection rate of depression symptoms was higher than that of anxiety symptoms. A previous study investigated 7,462,117 Chinese college students’ mental health problems, and the results showed that 21.1% of them were detected depression symptoms, 11% of them were detected anxiety symptoms, and the co-detection rate was 9.6%^22^. The detection rates of various items in this study were slightly higher than those of previous studies, which might result from the fact that this study was conducted in 2022 when the epidemic was severe, students' activity scope was limited, learning mode was changed, social and recreational activities were reduced, and students' negative emotions were serious^23^. In addition, different assessment tools used by researchers might also lead to differences in results.

The association between the specific symptoms of depression and anxiety among university students is the crux of this research area at the moment. Through canonical correlation analysis, this study verified the significant correlation between depression and anxiety, which was basically consistent with previous research results^24,25^, which might be related to the common genetic vulnerability of the depression and anxiety^26^. On the basis of supporting the results of previous studies, this study further found that depressive symptoms in male students might be synchronized with anxiety and motor tension of anxiety symptoms, while somatic disorders of depressive symptoms were closely related to psychomotor disorders, and affective disorders were closely related to depressive psychological disorders. The affective disorders and somatic disorders of female college students' depressive symptoms might be synchronized with anxiety, and the depressive psychological disorders might be synchronized with premonition of misfortune and frequent urination. This was an important supplement to the existing research, which could assist the diagnosis and classification of symptoms, and explore whether specific symptoms affect the prognosis.

This study demonstrated that physical exercise was negatively associated with the depression and anxiety symptoms among college students, which is consistent with the results of previous cross-sectional studies^27,28^. In particular, the college students with high levels of physical activity had lower depression and anxiety scale scores than the medium and low level groups, while there were no significant differences between the medium and low level groups, suggesting that more physical activity participation is required to reap emotional benefits (scores of all symptoms in the high-level group was significantly lower than that in the low level group). Previous evidence supports the results of this study that people with a higher level of physical activity had a 12–32% lower risk of developing depressive symptoms and a 15–34% lower risk of developing anxiety (Wolf et al.^29^). There was also a longitudinal observational study using the latent class growth model to follow up 1265 college students for 5 times, and the results showed that the state of continuous low exercise would increase the risk of high level depression and anxiety symptoms^30^, this conclusion was supported from another side. Different types of college students had significant differences in physical activity habits, and different physical activity habits were also associated with different risks of depression and anxiety, which provided a theoretical basis and breakthrough direction for exercise intervention in college students' mental health. A large number of previous studies have explored the mechanism of action, finding that physical activity can effectively regulate the concentration of neurotrophic factors, glucocorticoid levels and effect on neuroimmune system, induce hippocampal neurogenesis of the central nervous system, effectively stimulate the central nervous system, and then prevent and relieve depression and anxiety symptoms^16,31^. In addition, the improvement of self-efficacy^32^, social support^33^ and executive function^34^ caused by physical activity also have a positive impact on the mental health of college students.

More importantly, this study showed that the emotional benefits of physical activity were closely related to factors such as exercise intensity, duration and frequency, and there might be both a dose effect and a selective effect, which is similar to the conclusion of previous studies^35^.

As for exercise intensity, this study showed that high-intensity exercisers had the lowest risk of depression and anxiety, which might result from the fact that high-intensity exercise could stimulate body temperature rise more quickly. It could increase the secretion of endorphins^36^, stimulate the growth of serotonergic and dopaminergic neurons, and induce the increase of thalamic-cortical circuits, thus reducing the risk of anxiety and depression^37^. The removal of negative emotions in young people might require appropriate exercise intensity within the adaptive range of the body. Moderate-to-high intensity physical exercise is more effective than low-intensity physical exercise, which may not be enough to stimulate the neurological and hormonal changes that are associated with improvements in depression and anxiety^38,39^. Overall, our findings further support the physical activity guidelines recommended by the World Health Organization.

As for the duration of exercise, studies have shown that 20–60 min of exercise is the most beneficial, while over 75 min will have adverse effects on mood^40^. Exercise could promote the secretion of endorphins and dopamine, but there was a delay effect and threshold effect, so the exercise duration should not be too short. While exercise duration was too long, it would result in excessive fatigue and negative emotions. In this study, more than 60 min was recommended, perhaps because the self-rating scale was used in this study, participants would take into account the warm-up, interval and relaxation time after exercise, it is suggested that future research should further refine this.

As for the frequency of exercise, this study showed that a lower incidence of depression and anxiety symptoms was associated with physical activity more than three times a week. Convergent evidence showed that emotional benefits were generated immediately after exercise starts and were maintained for one day after stopping exercise^35^. Depression and anxiety symptoms can only be improved by exercising for more than three times a week, and it is best to do moderate exercise every day^41,42^. Therefore, it is recommended to insist on doing exercise every day.

## Conclusion or recommendation

Depression and anxiety symptoms of college students were closely related, and co-occurrence was common. Students with high level of physical activity had lower symptoms, the emotional benefits of physical activity were closely related to factors such as exercise intensity, duration and frequency, so there might be both a dose effect and a selective effect. Different exercise interventions are recommended for different symptoms.

## Limitations

1. The tools for measurements of this study are all self-reported questionnaires, which may have some subjective bias. It is suggested that accelerometers and heart rate bands should be used to evaluate physical activity in the future, and psychologist interview, EEG diagnosis as well as other methods should also be used to evaluate depression and anxiety symptoms.
2. It is suggested that longitudinal observation among college students should be conducted in the future, and the change trajectory of depression and anxiety symptoms in different groups should be distinguished through the latent class growth model, and the dose–effect and selective influence of physical activity through experiments should also be clarified.
3. This study did not discuss the relationship between various elements of physical activity and specific symptoms of depression and anxiety, which can be measured by subsequent experimental studies, which is conducive to the construction of accurate exercise programs.

## Data Availability

The datasets used and/or analysed during the current study available from the corresponding author on reasonable request.
